# Safety of COVID‐19 vaccination in children with prior allergic reactions or mast cell disorders

**DOI:** 10.1002/clt2.12267

**Published:** 2023-05-24

**Authors:** Rishabh Kulkarni, Annabelle Arnold, Adrian Buzynski, Michaela Lucas

**Affiliations:** ^1^ Immunology Department Sir Charles Gairdner Hospital Perth Western Australia; ^2^ Medical School University of Western Australia Perth Western Australia; ^3^ Immunology Department Perth Children's Hospital Perth Western Australia; ^4^ Immunology Department PathWest Laboratory Medicine Perth Western Australia

To the editor,

Over 13 billion Coronavirus disease 2019 (COVID‐19) vaccines have been administered worldwide. However, there is a lack of evidence‐based guidance on the management of children with reported allergic reactions to the COVID‐19 vaccine or those with pre‐existing conditions such as mast cell disorders.

To date, only one paper by Rosa Duque et al (2022),^1^ reported on three children between 12 and 17 years of Chinese and Indian ethnicity developing urticaria as their index reaction. They subsequently safely administered their second COVID‐19 vaccination after basophil activation testing with one child suffering mild urticaria after the challenge.^1^ The present recommendations suggest that patients who experience immediate allergic reactions to mRNA COVID‐19 vaccines should undergo skin testing for the vaccine or its excipients. A systematic review revealed that skin testing has low sensitivity but high specificity in predicting repeat immediate hypersensitivity reactions, but it is noteworthy that all re‐vaccinations were performed in adults.^2^

Given the high degrees of parental anxiety around childhood immunisation and the likelihood of seasonal vaccine boosters against emerging strains of COVID‐19, there is a need for more research on reported adverse reactions in children together with guidance on testing, management and public health messaging.

We present a series of paediatric cases who underwent testing and challenged currently available paediatric COVID‐19 vaccines to determine future safety of administration (Table 1). At this time, only two vaccines were approved in Australia for young children. Pfizer Comirnaty (BNT162b2) was approved for children over 5 years old on 3 December 2021 and Moderna Spikevax (mRNA 1273) in children over 6 years old on 17 February 2022. Ten Caucasian children between 8 and 15 years old, with either a reported history of allergy to COVID‐19 vaccines or with pre‐existing conditions which may have put them at a higher risk of a reaction, were referred to the Immunology service at Perth Children's Hospital, the sole tertiary paediatric hospital in Western Australia. Of these, three children had reported hypersensitivity reactions with cutaneous symptoms such as urticaria and angioedema, in one case combined with throat tightness, after their first mRNA COVID‐19 vaccination and three after both. Five had received Comirnaty and one had received Spikevax. Furthermore, two children had mastocytosis, one had mast cell activation syndrome and one suffered from idiopathic anaphylaxis managed on Omalizumab 150 mg fortnightly.

**TABLE 1:** Clinical details and outcomes.

Age (y)	Sex	Allergy history	Culprit vaccine	Number of vaccines prior to testing	Reported index reaction	Time following vaccine	Skin prick test	Intradermal test	Challenge
12	M	Nil	Comirnaty	1	Erythematous rash on trunk and legs and subjective throat tightness	<1 h	Negative	Negative	Successful Comirnaty challenge
13	M	Egg‐ anaphylaxis Cow milk‐ anaphylaxis	Spikevax	2	Injection arm urticaria	<1 h	Positive	Positive	Not challenged, fully vaccinated
12	M	Asthma Shellfish‐ anaphylaxis Bee venom‐ large local reaction Intermittent hay fever Influenza vaccine‐ urticaria	Comirnaty	2	Injection arm urticaria	<1 h	Negative	Negative	Not challenged; fully vaccinated
13	F	HPV vaccine‐ anaphylaxis	Comirnaty	2	Injection arm urticaria	>4 h	Negative	Negative	Not challenged; fully vaccinated
11	F	Cats‐ allergic rhinoconjunctivitis	Comirnaty	1	Facial urticaria and angioedema	>1–<4 h	Negative	Negative	Successful Comirnaty challenge
8	F	Nil	Comirnaty	1	Generalised urticaria	<1 h	Negative	Equivocal to Comirnaty; negative to Spikevax	Successful Spikevax challenge
14	M	Mastocytosis	N/A	0	N/A	N/A	N/A	N/A	Successful Comirnaty challenge
13	F	Mastocytosis	N/A	0	N/A	N/A	N/A	N/A	Successful Comirnaty challenge
14	F	Mast cell activation syndrome	N/A	0	N/A	N/A	N/A	N/A	Successful Comirnaty challenge
15	F	Idiopathic anaphylaxis on omalizumab 150 mg fortnightly	N/A	0	N/A	N/A	N/A	N/A	Successful Comirnaty challenge

The six children with reported reactions had skin‐prick testing (SPT) and intradermal testing (IDT) followed by a graded vaccine challenge as is standard protocol in our centre for suspected IgE‐mediated reactions. The SPT used sodium chloride 0.9% as a negative control and histamine as a positive control with standard EAACI definitions used to determine positive (wheal diameter >3 mm) and negative results.^3^ If negative, the patient proceeded to IDT. This was done in two steps using sodium chloride 0.9% as a negative control, along with 0.02 mLs of 1/100^th^ of the vaccine initially, and if negative, followed by 0.02 mL of 1/10^th^ of the relevant vaccine in Step 2. SPT and IDT were read at 15 min.

After negative skin testing, a graded vaccine challenge at 30‐min intervals was performed starting with 10% of the vaccine dose followed by the remaining 90%. Patients were monitored for 1 hour with regular observations and then followed up by telephone on days 2 and 7 to ensure no delayed reactions. The four patients with pre‐existing conditions were directly challenged under medical supervision without skin testing.

Of the six children who were skin tested, four had negative skin testing; two of these four had a successful challenge to the culprit vaccine. Two were not challenged as they were already fully vaccinated according to current guidelines. One of the six had an equivocal skin test for Comirnaty but was negative for Spikevax and underwent a successful Spikevax challenge without adverse events. The remaining child had a positive skin test for both Comirnaty and Spikevax; he had received two Comirnaty vaccines in the community and had similar mild localised cutaneous reactions on both occasions. He was negative for Novavax (NVX‐CoV2373) on skin testing. He was not challenged as had completed his primary vaccine course but was advised to avoid mRNA vaccines with the potential to have Novavax in the future. The four children with pre‐existing conditions had successful challenges with no adverse events. No one was premedicated prior to challenge and there were no delayed reactions.

Our data show that 9 of 10 children studied would be safely able to have at least one brand of mRNA COVID‐19 vaccine in the future. Patients were not challenged with non‐mRNA vaccines as they were not licensed at the time, but as of July 2022, Novavax gained approval in children over 12 years.

Results from this paediatric cohort are consistent with studies in adults who tolerated second doses of the vaccines despite adverse reactions to the first.^4^ Reasons might include non‐IgE mediated mechanisms such as activation of complement pathways, direct mast cell activation, and contact system activation by RNA.^5^ Furthermore, although patients with mast cell activation syndromes have been shown not to be at higher risk of allergic reactions to vaccines or drugs, they are frequently subjected to pre‐medication and very few children are included in these studies. In our cohort consisting solely of paediatric patients, those with mast cell disorders safely tolerated the COVID‐19 vaccine without premedication replicating previous findings seen where premedication was used.^6^, ^7^

Despite the low incidence rate of severe allergic reactions, there is significant anxiety within the general population. Twelve percent of parents who chose not to vaccinate their children cited fears of an allergic reaction.^8^ Despite Australia being a highly vaccinated country, with over 95% of adults being fully vaccinated for COVID‐19, as of December 2022 only 49% of children aged 5–11 years, and 74% of children aged 11–15 years had received one dose of the vaccine^9^ compared to greater than 95% immunisation rate against MMR and Polio.^10^, ^11^

More global paediatric data on allergic and adverse reactions to COVID‐19 vaccines and trials on optimal management might help improve immunisation rates in children and alleviate parental fears.

## AUTHOR CONTRIBUTIONS

**Rishabh Kulkarni**: Formal analysis (Equal); Writing – original draft (Lead); Writing – review & editing (Lead). **Annabelle Arnold**: Conceptualization (Equal); Resources (Equal); Writing – original draft (Supporting); Writing – review & editing (Supporting). **Adrian Buzynski**: Conceptualization (Equal); Formal analysis (Equal); Methodology (Equal); Resources (Equal); Writing – original draft (Supporting); Writing – review & editing (Supporting). **Michaela Lucas**: Conceptualization (Equal); Formal analysis (Equal); Resources (Lead); Supervision (Lead); Writing – original draft (Equal); Writing – review & editing (Equal).

## CONFLICT OF INTEREST STATEMENT

There are no funding sources to declare and the authors declare that they have no relevant conflicts of interest.
